# Perioperative and Long-Term Outcomes of Acute Stanford Type A Aortic Dissection Repair in Octogenarians

**DOI:** 10.3390/medsci12030045

**Published:** 2024-09-02

**Authors:** Hannah Masraf, Manoraj Navaratnarajah, Laura Viola, Davorin Sef, Pietro G. Malvindi, Szabolcs Miskolczi, Theodore Velissaris, Suvitesh Luthra

**Affiliations:** 1 Division of Surgery, Kingston Hospital NHS Foundation Trust, Kingston upon Thames KT2 7QB, UK; 2 Wessex Cardiothoracic Centre, Division of Cardiac Surgery, Southampton University Hospital NHS Foundation Trust, Southampton SO16 6YD, UK; 3 Cardiac Surgery Unit, Lancisi Cardiovascular Center, Ospedali Riuniti delle Marche, Polytechnic University of Marche, 60126 Ancona, Italy; 4 Department of Human Development and Health, Faculty of Medicine, University of Southampton, Southampton, SO17 1BJ, UK

**Keywords:** acute aortic dissection, type A aortic dissection, octogenarians, Stanford type A, aortic dissection repair

## Abstract

Background: The aims of this study were to assess the perioperative morbidity, mortality and long-term survival of octogenarians undergoing acute type A aortic dissection repair (ATAAD), and to compare open and closed distal anastomosis techniques. Methods: This was a single-centre retrospective study (2007–2021). Open versus closed distal anastomosis were compared. Uni- and multivariable logistic regression analyses were performed to identify independent predictors of in-hospital mortality. Kaplan–Meier and Cox proportional hazards methods were used to compare long-term survival. Results: Fifty octogenarian patients were included (median age—82 years; closed distal—22; open distal—28). Median cardiopulmonary bypass time was 187 min (open distal vs. closed distal group; 219 min vs. 115.5 min, *p* < 0.01, respectively). Median cross-clamp time was 93 min (IQR; 76–130 min). Median circulatory arrest time was 26 min (IQR; 20–39 min) in the open-distal group. In-hospital mortality was 18% (open distal; 14.2% vs. closed distal; 22.7%, *p* = 0.44). Stroke was 26% (open distal; 28.6% vs. closed distal; 22.7%, *p* = 0.64). Median survival was 7.2 years (IQR; 4.5–11.6 years). Survival was comparable between open and closed distal groups (median 10.6 vs. 7.2 years, *p* = 0.35, respectively). Critical preoperative status (HR; 3.2, *p* = 0.03) and composite endpoint (renal replacement therapy, new neurological event, length of stay > 30 days or return to theatre; HR; 4.1, *p* = 0.02) predicted adverse survival. Open distal anastomosis did no impact survival. Conclusions: ATAAD repair in selected octogenarians has acceptable short- and long-term survival. There is no significant difference between open versus closed distal anastomosis strategies.

## 1. Background

The United Kingdom (UK) population is ageing, with >15.5 million people over 60 years old, and 3.2 million octogenarians (4.7%) [1]. The largest increase in population is predicted to be in the older age groups while the fraction of people over 85 years is predicted to double to 3 million by 2041. The proportion of octogenarians undergoing coronary artery bypass grafting has risen threefold to 7.2% in UK from 2002 to 2016 [2]. With increasing life expectancy and an increasing proportion of elderly patients fit for cardiac surgery and cardiology interventions, the number of octogenarians with acute Stanford type A aortic dissection (ATAAD) requiring operative intervention is expected to rise over the coming decades. This may also include a new class of more frequent iatrogenic dissections [3]. Outcomes following surgical ATAAD repair have continued to improve over the last decade, with large volume aortic centres consistently reporting mortalities of <10%. However, age is perceived as a negative survival predictor and a deterrent for surgical intervention due to poor outcomes. A resource- and cost-intensive, high-risk emergency surgery for octogenarians remains controversial from a medical and social perspective in overstretched health care systems. The aims of this study were to assess the perioperative morbidity, mortality and long-term survival outcomes of octogenarians undergoing ATAAD repair, and to compare open and closed distal anastomosis surgical repair techniques.

## 2. Methods

### 2.1. Study Design

This was a single-centre retrospective study (2007–2021) performed at the University Hospital Southampton (UHS). Data for all octogenarian patients who underwent surgical repair of AAATD were collected from the hospital database (Patient Administration System—PAS), e-CAMIS (Electronic Clinical and Management Information System, Yeadon, Leeds, UK). Institutional approvals were obtained for the use of data in compliance with the local data protection policies (safeguard approval number 7373, 15 November 2022). Consent for individual use of data was waived due to the retrospective nature of this study and prior approvals at the time of consent for procedures. Inclusion criteria included all octogenarians who underwent emergency/salvage ATAAD repair within 14 days of onset of symptoms. Iatrogenic and intraoperative aortic dissections during cardiac surgery were included. Patients who died without definitive surgery (at or prior to anaesthetic induction or prior during the transfer) were excluded.

### 2.2. Surgical Techniques

ATAAD was confirmed by preoperative computed tomography in all cases and echocardiography was performed intraoperatively. Surgeons participating in the aortic dissection rota performed the procedures. Cannulation technique, cerebral protection strategy, open versus closed distal anastomosis and degree of hypothermia were selected primarily by the surgeon’s preference and their individual techniques. Generally, frailty, presentation, avoidance of deep hypothermic arrest and circulatory arrest would dictate the choice between open and closed anastomosis. Our standard surgical procedure consisted of median sternotomy with standard CPB established via femoral/axillary arterial cannulation with systemic hypothermia. Myocardial protection was achieved with antegrade or retrograde cold blood cardioplegia at 4 °C. All repairs included replacement of the ascending aorta using an interposition graft with open or closed distal anastomosis, with or without extension into the arch/hemiarch and any other concomitant procedures deemed necessary. A tear-oriented surgical strategy was employed. Ascending aortic replacement was performed if the primary tear was in the ascending aorta or in the case it was not identified. Extended aortic resection and repair (hemiarch/arch) were performed if an arch tear was identified. ATAAD involving aortic root was either repaired using BioGlue or aortic root repair/replacement was performed. All open distal procedures were performed under deep hypothermic circulatory arrest (DHCA) at 24–28 °C (nasopharyngeal) with/without antegrade selective/nonselective cerebral perfusion depending on cannulation strategy. Near-infrared spectroscopy (NIRS) was used in all cases for neurocerebral monitoring. In the case of significant bilateral discrepancy between NIRS readings, a selective cannulation strategy through the arch was used for cerebral perfusion.

### 2.3. Data Collection

Baseline demographics included data used for the NICOR (National Institute of Cardiac Outcomes Research, UK) database (Table 1). Critical preoperative status was defined as tamponade, cardiac arrest, end organ ischemia (myocardial infarction, gut infarction or stroke) or need for ventilatory or haemodynamic support (infusion of vasopressors or inotropes). Operative and postoperative data included type (open versus closed distal anastomosis) and extent of surgery, other concomitant procedures, cannulation technique, cardiopulmonary bypass time (CPB), cross-clamp time (XCT), total circulatory arrest time (TCA) and neuroprotection strategy. Postoperative data included return to theatre, new cerebrovascular accident (CVA), length of hospital stay (LOS), renal replacement therapy (RRT), in-hospital mortality and a composite endpoint consisting of RRT, new CVA, LOS ≥ 30 days, re-exploration and in-hospital mortality. TIA was defined as transient neurological dysfunction of less than 24 h, and stroke of >24 h. The neurological dysfunction was always reviewed by a neurologist and confirmed radiologically on a CT scan.

Long-term survival was obtained from PAS, e-CAMIS and the electronic database of General Practitioners’ medical records linked to the hospital database on National Healthcare Service Spine Portal Summary Care Records (SCR) system.

### 2.4. Statistical Analysis

All clinical data were analysed retrospectively through a review of prospectively collected electronic and archived medical records. Postoperative survival was calculated from the time of surgery to death from any cause. Continuous variables are presented as mean ± standard deviation. Categorical variables are presented as counts and percentages. Categorical data were compared with the chi-square test whereas continuous data were compared with Student’s *t*-test. Demographic data and operative strategies of open versus closed distal anastomosis were compared including preoperative variables. Uni- and multivariable logistic regression analyses were performed to identify independent predictors of in-hospital mortality. Variables with a *p*-value < 0.05 were entered into a multivariable model. Kaplan–Meier and Cox proportional hazards methods were used to compare long-term survival. Statistical analyses were performed using Stata statistical software v17 (StataCorp. College Station, TX, USA, 2021). A *p*-value of <0.05 was considered statistically significant.

## 3. Results

### 3.1. Preoperative Characteristics

Fifty octogenarians underwent surgery for ATAAD (22 with closed distal and 28 with open distal anastomosis). Median age was 82 years (IQR; 80.3–83.4 years), with female predominance (58%). Five (10%) patients presented with iatrogenic ATAAD either during cardiac catherisation or intraoperatively during cardiac surgery. There were no significant differences in preoperative characteristics between patients with open or closed distal anastomosis (Table 1). However, the closed distal group had more commonly preoperative neurological deficit as compared to open distal group (9.3% vs. 0%, *p* = 0.10, respectively). Patients in the open distal group had more commonly extracardiac arteriopathy (14.3% vs. 4.5%, *p* = 0.25), cardiogenic shock (32.1% vs. 13.6%, *p* = 0.13) and lower preoperative haemoglobin 120 vs. 127 g/L, *p* = 0.05). The closed distal group had a higher proportion of patients with previous sternotomy as compared to the open distal group, although the difference was not statistically significant (13.6% vs. 3.6%, *p* = 0.19, respectively).

### 3.2. Operative Characteristics

Operative characteristics are demonstrated in Table 2. Femoral artery cannulation was performed most commonly (62%). Median CPB time was 187 min (IQR; 121–245 min). CPB time was significantly higher in the open distal as compared to the closed distal group (median 219 min vs. 115.5 min, *p* < 0.01, respectively). Median cross-clamp time (XCT) was 93 min (IQR; 76–130 min). Median TCA time was 26 min IQR; 20–39 min) in the open-distal group.

Nonselective antegrade cerebral circulation was used for neuroprotection in 22% of all cases and 35.7% of all open-distal anastomosis cases. Concomitant procedures were performed in additional 28%.

### 3.3. Postoperative Characteristics

In-hospital mortality was 18% (14.2% in the open distal vs. 22.7% in the closed distal group, *p* = 0.44). The mortality associated with iatrogenic dissections was higher (4/5, 80%) than for spontaneous dissections (5/45, 11.1%).

We observed higher postoperative CVA rate in the open distal group (39.3% vs. closed distal, 22.7%, *p* = 0.21). Intraoperatively, three patients (6%) died after failure to wean off CPB. All these three patients had iatrogenic ATAAD (cardiac catherisation, intraoperative mitral valve replacement and a redo sternotomy) with involvement of the coronary ostia. There was no significant difference in the composite endpoint of RRT, CVA, LOS ≥ 30 days, re-exploration or in-hospital mortality (open distal 45.5% vs. closed distal 57.1%, *p* = 0.41) (Table 2). LVEF < 30, NHYA class 3/4, XCT, LOS, preoperative creatinine, CPB time, concomitant cardiac procedure and male gender were independent predictors of in-hospital mortality on univariate regression, but not multivariate regression (Table 3).

**Figure 1:**
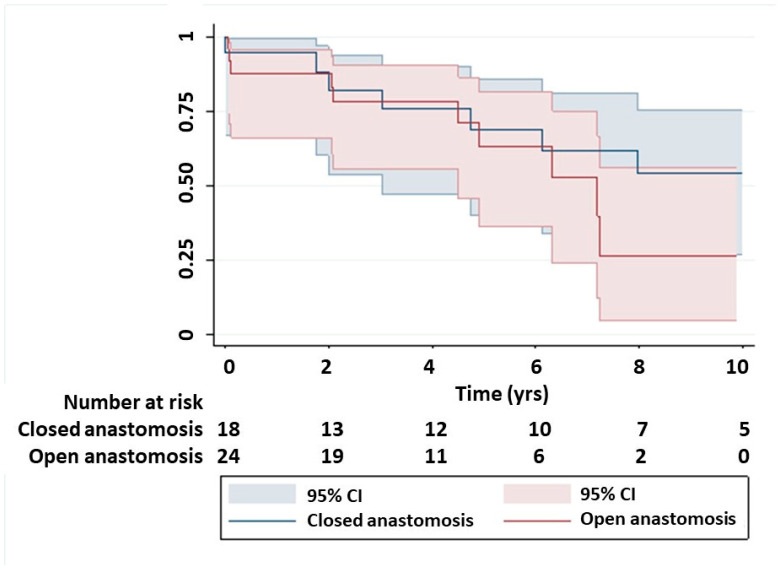
Kaplan–Meier survival curves comparing open vs. closed distal anastomosis technique.

The mean follow-up was 8.5 years, and the completion of follow-up was 100%. Median survival was 7.2 years (IQR; 4.5–11.6 years). Long-term survival was comparable between open and closed distal groups (median 10.6 vs. 7.2 years, *p* = 0.35, respectively) (Figure 1). Critical preoperative status (preoperative neurological deficit, preoperative renal failure, requirement for intravenous inotropes, cardiogenic shock or preoperative mechanical ventilation; HR; 3.2, *p* = 0.03) and composite endpoint (RRT, new CVA, LOS > 30 days or return to theatre; HR; 4.1, *p* = 0.02) were predictors for adverse long-term survival (Table 4). Open distal anastomosis did not impact survival.

Thirty-four percent of the patients were discharged home, and the others were sent to a convalescent home (32%) or another hospital (16%) for recuperation. Survival was 75.8 ± 6.1% at 1 year (76.7% for closed distal vs. 75.0% for open distal anastomosis, *p* = 0.16) and 55.1 ± 7.7% (7.7%) at 5 years (56.3% for closed distal vs. 52.9% for open distal anastomosis, *p* < 0.01).

## 4. Discussion

International Registry for Aortic Dissection (IRAD) data demonstrated that surgical repair of ATAAD in octogenarians is associated with improved 5-year survival when compared to medical treatment [4]. IRAD data showed that early postoperative mortality rates appeared similar between septuagenarians and octogenarians (25.1% vs. 21.7%, *p* = 0.205), yet a significantly smaller proportion of octogenarians were operated upon (68.1% vs. 85.9%, *p* < 0.001). Furthermore, comparable rates of postoperative complications demonstrated that surgical repair in selected octogenarians can provide satisfactory outcomes [5,6]. In our series, we demonstrated satisfactory in-hospital mortality and 5-year survival among octogenarians undergoing ATAAD repair, and our results were comparable to the IRAD data. However, octogenarians still represent a high-risk group, and reported in-hospital mortality after ATAAD repair is generally higher than in younger patients [7,8,9,10,11,12]. Another important finding was that critical preoperative status including preoperative neurological deficit, renal failure, requirement for intravenous inotropes, cardiogenic shock or mechanical ventilation were factors negatively associated with long-term survival of octogenarians (HR; 3.2, *p* = 0.03).

Open distal anastomosis is advocated as the preferred surgical technique [13]. It allows for resection of much of the dissected aorta, an easier distal anastomosis even into the hemiarch/arch and arch tears can be inspected. Since prolonged surgery and CPB time may be deleterious in octogenarians, conservative procedures including closed distal anastomosis may be believed to improve outcomes. However, we have previously shown that there was no difference in late survival between patients receiving an open or a closed distal anastomosis [14]. Our mean CPB times and XCT times were comparable to IRAD data: (181.0 min (142.3–236.8) and 117.0 min (87.0–156.0), respectively). Although CPB times were significantly longer in our open distal anastomosis group, this did not increase in-hospital mortality (14.2% in open distal vs. 22.7% in closed distal anastomosis group). The 5-year survival was comparable in both groups. Such findings suggest that open distal anastomosis with extended resection into the arch in selected octogenarian patients can be performed with satisfactory outcomes. On the other hand, a strategy to perform a quicker and less complex operation may be advisable in high-risk octogenarians in compromised circumstances of haemodynamic instability, malperfusion or general debility and frailty.

Interestingly, octogenarians account for up to 20% of patients undergoing ATAAD repair in Japan due to an elderly population. Several Japanese studies consistently showed low mortality rates (4.8–14.3%) and excellent outcomes in octogenarians with both conservative and extended resection techniques [15,16,17,18]. Data from the Japanese Registry of Aortic Dissection (JRAD) for 2011–2016, however, showed that age older than 80 years was a risk factor for in-hospital mortality (odds ratio, 2.37; *p* < 0.01) [19]. Other studies, outside of Japan, have shown good outcomes of ATAAD repair in octogenarians albeit with higher mortality compared to non-octogenarians [20,21]. A large meta-analysis of 16 retrospective studies by Eranki et al. with 16,641 patients showed that octogenarians displayed significantly higher in-hospital mortality than non-octogenarians (OR 1.93; 95% CI 1.33–2.81; *p* < 0.001) [22]. The 5-year survival in the octogenarian cohort of 54% was significantly lower compared to 76% in the non-octogenarian cohort (*p* < 0.001). Similarly, the meta-analysis by Biancari et al. showed in-patient mortality of 18.4% (4–56%)—20.7% in non-Japanese studies (7–56%) and 16.3% in Japanese studies (4–21%).

Concerns regarding quality of life (QOL) and resource utilisation after ATAAD repair means that octogenarians are more likely to be managed conservatively [23]. Mean hospital LOS in our study was 16.5 days, which is longer than that reported by IRAD (13.0 days). This may represent differences in hospital and intensive care unit discharge policies and country-based discharge policies, and detailed comparison was not possible. Wide variation is reported in the international literature ranging from hospital LOS of 12–41 days. Although there is an argument for a simpler, more conservative repair technique with a closed distal anastomosis in the elderly to expedite postoperative recuperation, we did not observe a significant difference in LOS in the open distal as compared to the closed distal anastomosis group (14.6 days versus 13.8 days, *p* = 1.00). Ghazy et al. demonstrated that an aggressive surgical strategy did not improve the quality of life in midterm follow-up compared to a defensive strategy [24]. In their short prospective study of 39 patients, a “defensive” strategy had shorter operative times (184 ± 54 versus 276 ± 110 min, *p* = 0.001) and superior mid-term QOL scores. Only 34% of our patients were fit enough to be discharged home and others were discharged to a convalescence home or another hospital for ongoing recuperation. There was no difference between the surgical strategies.

Iatrogenic dissections from cardiac catherisation and intraoperatively are not uncommon in octogenarians. Although no data exist about incidence of these dissections, these are likely to be more common with increased interventions (both cardiological and vascular catherisations and interventions and cardiac surgeries in elderly). These are also likely to carry a worse prognosis as a result of an added life-threatening complication of another major procedure in elderly high-risk patients.

Evidence is growing that QOL after surgery is significantly diminished, particularly in the elderly compared to age-matched general population [25,26,27]. However, equivalent mid-term QOL scores between octogenarian and younger non-octogenarians undergoing ATAAD repair have also been demonstrated. Few studies have precisely addressed QOL following ATAAD repair. Well-designed prospective studies using standardised QOL questionnaires, are needed to make reliable conclusions on the health-related QOL outcomes following ATAAD in octogenarians. This will better guide future appropriate management in this high-risk population.

## 5. Limitations

This study has the inherent limitations of a retrospective, single-centre study, and is limited by a relatively small sample size and wide confidence intervals. Because of the limited number of patients and perioperative events, variables for multivariable models were also limited. Results should therefore be cautiously interpreted. Inherent selection bias may confound results, as only those who were offered surgery were evaluated. Those who died at transfer or were moribund and not offered surgery were excluded from this analysis. Direct comparison with results of a younger non-octogenarian cohort may have provided better perspective for outcomes. Unfortunately, further comparisons between surgical techniques, extent of surgical repair, cannulation technique and neurocerebral protection strategies were not possible due to small number of patients. Lastly, postoperative QOL and level of physical activity was not evaluated quantitatively and merits further future study since these are critical factors in the ongoing debate surrounding appropriateness of intervention for ATAAD in the elderly.

## 6. Conclusions

Despite relatively high-risk of morbidity and mortality, ATAAD repair in selected octogenarians has acceptable short- and long-term survival and surgical repair may be justified. Age per se should not be considered an exclusion criterion for surgical repair of ATAAD. There is no significant difference in short- and long-term survival of octogenarians between open versus closed distal anastomosis strategies.

## Figures and Tables

**Table 1:** Preoperative demographic and clinical characteristics.

Variable	Total	Closed (n = 22)	Open (n = 28)	*p* Value
Age, years (IQR)	82 (80.3–83.4)	82.8 (80.3–85)	81.9(80.4–82.9)	0.23
Male gender	21 (42%)	9 (40.9%)	12 (42.9%)	0.89
Log EuroSCORE, % (IQR)	40.6 (35.6–59.2)	40.6 (35.9–57.3)	39.8 (34.3–59.8%)	0.95
Angina class 3–4	11 (22%)	4 (18.2%)	7 (25%)	0.56
NYHA class 3–4	3 (6%)	0 (0%)	3 (10.7%)	0.11
Previous cardiac surgery	4 (8%)	3 (13.6%)	1 (3.6%)	0.19
Previous MI	2 (4%)	1 (4.5%)	1 (3.6%)	0.86
Diabetes mellitus	2 (4%)	1 (4.5%)	1 (3.6%)	0.86
Hypertension	36 (72%)	16 (72.7%)	20 (71.4%)	0.92
Smoking history	15 (30%)	7 (31.8%)	8 (28.6%)	0.80
Preop renal failure	3 (6%)	1 (4.5%)	2 (7.1%)	0.70
Preop Hb, g/L (IQR)	120 (110–133)	127 (113–137)	120 (110–124)	0.05
Preop COPD	5 (10%)	2 (9.1%)	3 (10.7%)	0.85
Preop neurological deficit	2 (4%)	2 (9.1%)	0 (0%)	0.10
Extracardiac arteriopathy	5 (10%)	1 (4.5%)	4 (14.3%)	0.25
LVEF ≤ 30	2 (4%)	1 (4.5%)	1 (3.6%)	0.86
Cardiogenic shock	12 (24%)	3 (13.6%)	9 (32.1%)	0.13
Preop inotropes	3 (6%)	1 (4.5%)	2 (7.1%)	0.70
Cause of dissection				
Hypertension	44 (88%)	19 (86.4%)	25 (89.3%)	
Aneurysm	1 (0.2%)	1 (0.5%)	0	
Iatrogenic	5 (10%)	2 (0.9%)	3 (10.7%)	

Data are presented as median (quartiles; minimum–maximum) or count (percent). Abbreviations: NYHA—New York Heart Association; MI—myocardial infarction; COPD—chronic obstructive pulmonary disease; Hb—haemoglobin; LVEF—left ventricular ejection fraction; LOS—length of stay.

**Table 2:** Operative and postoperative characteristics.

Variable	Total (50)	Closed (n = 22)	Open (n = 28)	*p* Value
OPERATIVE CHARACTERISTICS				
Cannulation technique				
Femoral	31 (62.0%)	14 (63.6%)	17 (60.7%)	
Subclavian	10 (20.0%)	3 (13.6%)	7 (25.0%)	
Central	7 (14.0%)	3 13.6%)	4 (14.3%)	
Unknown	2 (4.0%)	2 (9.1%)	0	
XCT, min (IQR)	93 (76–130)	81 (69–112)	101 (79–146)	0.31
CPB, min (IQR)	187 (121–245)	115.5 (102–205)	219 (184–282)	<0.01
TCA, min (IQR)	n/a	n/a	26 (20–39)	
Neuroprotection				
Antegrade	11 (22%)	1 (4.6%)	10 (35.7%)	
Retrograde	3 (6.0%)	0	3 (10.7%)	
None	36 (72%)	21 (95.4%)	15 (53.6%)	
Aortic procedures				
1. Interposition tube graft without extension into the arch	31 (62%)	19 (86.4%)	12 (42.9%)	
2. Interposition tube graft with extension into the arch	13 (26%)	1 (0.45%)	12 (42.9%)	
3. Interpositional graft + separate valve	6 (12%)	2 (0.9%)	4 (14.3%)	
Other concomitant procedures				
1. Aortic valve/root repair/replacement	10 (20%)	5 (22.7%)	5 (17.9%)	0.28
2. Coronary artery bypass	4 (8%)	2 (9.1%)	2 (7.1%)	
POSTOPERATIVE CHARACTERISTICS				
Return to theatre	3 (6%)	1 (4.5%)	2 (7.1%)	0.70
New TIA	3 (6%)	0 (0%)	3 (10.7%)	0.11
New stroke	13 (26%)	5 (22.7%)	8 (28.6%)	0.64
New ischemic neurological events	16 (32%)	5 (22.7%)	11 (39.3%)	0.21
RRT	4 (8%)	2 (9.1%)	2 (7.1%)	0.80
LOS, days (IQR)	14.6 (8.0–20.7)	13.8 (3.3–19.4)	14.6 (8.8–20.9)	1.00
LOS ≥ 30 days	5 (10%)	1 (4.5%)	4 (14.3%)	0.25
In-hospital mortality	9 (18%)	5 (22.7%)	4 (14.3%)	0.44
Composite endpoint *	26 (52%)	10 (45.5%)	16 (57.1%)	0.41
Discharge destination				
Home	17 (34%)	7 (31.8%)	10 (35.7%)	
Convalescence	16 (32%)	7 (31.8%)	9 (32.1%)	
Other hospital	8 (16%)	3(13.6%)	5 (17.9%)	
Median survival, years (IQR)	7.2 (4.5–11.6)	10.6 (4.7–11.6)	7.2 (4.5–8.1)	0.35
Survival				
6 months	76 ± 6.0%	77.3 ± 8.9%	85.7 ± 6.6%	0.08
1 year	75.8 ± 6.1%	76.7 ± 9.1%	75.0 ± 8.2%	0.61
5 years	55.1 ± 7.7%	56.3 ± 11.0%	52.9 ± 11.2%	0.18

Data are presented as median (IQR—interquartile range) or count (percent). Abbreviations: CBP—cardiopulmonary bypass time; CVA—cerebrovascular accident; TCA—total circulatory arrest time; LOS—length of stay; RRT—renal replacement therapy; TIA—transient ischemic attack; XCT—cross clamp time. * Composite endpoint of RRT, new CVA, LOS ≥ 30 days, return to theatre, in-hospital mortality.

**Table 3:** Logistic regression model for predictors of in-hospital mortality (<0.05 entered into the multivariable model).

	Univariable				Multivariable		
Variable	Odds Ratio (95% CI)	95% Confidence Interval	*p* Value	Included in Multivariable Model?	Odds Ratio (95% CI)	95% Confidence Interval	*p* Value
**Preoperative**							
Age	1.03	0.75, 1.40	0.87	N			
Female gender	0.18	0.035, 0.84	0.03	Y	0.35	0.03–3.5	0.37
Log EuroSCORE	9.69	0.20, 458.1	0.25	N			
Angina class 3–4	1.14	0.23, 5.67	0.87	N			
NHYA class ≥ 3	9.0	1.03, 78.7	0.05	Y	0.24	0.004, 14.8	0.50
Previous cardiac surgery	3.24	1.11, 9.49	0.03	Y	1.4	0.35, 5.8	0.62
Diabetes mellitus	4.76	0.44, 51.52	0.20	N			
Hypertension	2.68	0.42, 17.12	0.30	N			
Smoking history	0.81	0.25, 2.70	0.74	N			
Preop creatinine	1.02	1.00, 1.03	0.05	Y	1.03	0.99, 1.1	0.17
Preop haemoglobin	1.01	0.97, 1.06	0.62	N			
Preop COPD	3.67	0.61, 22.22	0.16	N			
Preop neurology Hx	0.83	0.04, 18.8	0.91	N			
Extracardiac arteriopathy	1.47	0.20, 10.78	0.70	N			
LVEF < 30	27.67	1.20, 635.62	0.04	Y	1.39	0.03, 81.2	0.87
Critical preop state *	1.27	0.30, 5.46	0.75	N			
**Operative**							
Additional procedures	6.68	1.49, 29.93	0.01	Y	1.41	0.12, 15.8	0.78
Open distal anastomosis	0.58	0.15, 2.34	0.45	N			
CPB time (mina)	1.01	1.00, 1.03	0.01	Y	1.01	0.99, 1.03	0.44
**Postoperative**							
Re-exploration for bleeding	0.58	0.03, 13.19	0.72	N			
RRT	5.27	0.77, 35.89	0.09	N			
New CVA	0.63	0.13, 3.03	0.57	N			
Composite endpoint	0.75	0.18, 3.17	0.70	N			
LOS	0.85	0.76, 0.96	0.01	Y	0.88	0.75, 1.03	0.12

Abbreviations: COPD—chronic obstructive pulmonary disease; LVEF—left ventricular ejection fraction; CPB—cardiopulmonary bypass time; CVA—cerebrovascular accident; LOS—length of stay; NYHA—New York Heart Association; RRT—renal replacement therapy; * Critical preop: preoperative neuro deficit, preoperative renal failure, inotropes, shock or mechanical ventilation.

**Table 4:** Cox regression model for predictors of long-term survival.

Variable	Hazard Ratio	95% CI	*p*-Value
Critical Preop ^a^	3.17	1.1, 8.9	0.03
Open distal anastomosis	1.00	0.3, 3.1	1.00
Concomitant procedure	1.30	0.4, 4.3	0.67
Composite endpoint ^b^	4.06	1.3, 12.7	0.02
Hypertension	0.40	0.1, 1.1	0.08

^a^ Critical preop: preoperative neuro deficit, preoperative renal failure, inotropes, shock or mechanical ventilation. ^b^ Composite endpoint of RRT, new CVA, LOS ≥ 30 days, return to theatre.

## Data Availability

Data are available for review on request, subject to institutional data protection policies.
